# Comparative effectiveness of tirzepatide versus GLP-1 receptor agonists on the risk of venous thromboembolism in patients with obesity: a real-world cohort study

**DOI:** 10.3389/fmed.2026.1820366

**Published:** 2026-05-19

**Authors:** Jheng-Yan Wu, Keng-Wei Lee, Sheng-Chi Huang, Hsuan-Yuan Chang, Yu-Min Lin

**Affiliations:** 1 Department of Nutrition, Chi Mei Medical Center, Tainan, Taiwan; 2 Department of Public Health, College of Medicine, National Cheng Kung University, Tainan, Taiwan; 3 Division of Cardiology, Department of Internal Medicine, Chi Mei Medical Center, Chiali, Tainan, Taiwan; 4 Department of Medical Education, Chi Mei Medical Center, Tainan, Taiwan; 5 Division of Hepatogastroenterology, Department of Internal Medicine, Chi Mei Medical Centre, Tainan, Taiwan; 6 Division of Cardiology, Department of Internal Medicine, Chi Mei Medical Center, Tainan, Taiwan

**Keywords:** deep vein thrombosis, glucagon-like peptide-1 receptor agonists, obesity, pulmonary embolism, tirzepatide, venous thromboembolism

## Abstract

**Background:**

Venous thromboembolism (VTE), including deep vein thrombosis (DVT) and pulmonary embolism (PE), represents a major cause of morbidity and mortality in patients with obesity. Although glucagon-like peptide-1 receptor agonists (GLP-1 RAs) have been associated with weight loss and potential reductions in VTE risk, evidence regarding tirzepatide remains limited. This study aimed to evaluate the comparative effectiveness of tirzepatide versus GLP-1 RAs in reducing the risk of VTE in patients with obesity.

**Methods:**

We conducted a retrospective cohort study using the TriNetX global federated health research network. Adults with obesity (body mass index ≥30 kg/m^2^ or ICD-10-CM diagnosis codes) who initiated tirzepatide or GLP-1 RAs between January 2022 and August 2025 were identified. An active-comparator, new-user design was applied. Propensity score matching (1:1) was used to balance baseline demographics, comorbidities, and laboratory parameters. The primary outcome was 1-year incidence of VTE; secondary outcomes included PE, DVT, and all-cause mortality. Negative control outcomes and E-values were used to assess residual confounding.

**Results:**

After matching, 701,374 patients were included. Tirzepatide initiation was associated with a significantly lower risk of VTE compared with GLP-1 RAs (hazard ratio [HR], 0.90; 95% confidence interval [CI], 0.83–0.98; *p* = 0.011). The benefit was driven by reductions in PE (HR, 0.88; 95% CI, 0.79–0.98; *p* = 0.021) and all-cause mortality (HR, 0.90; 95% CI, 0.82–0.99; *p* = 0.024), whereas no significant difference was observed for DVT (HR, 0.96; 95% CI, 0.86–1.07; *p* = 0.410).

**Conclusion:**

In this large real-world cohort, tirzepatide use was associated with a significantly lower 1-year incidence of VTE compared with GLP-1 RAs in patients with obesity, particularly through reductions in PE and all-cause mortality. These findings suggest an association between tirzepatide use and a lower risk of VTE compared with GLP-1 receptor agonists. However, given the observational nature of this study, causality cannot be established, and the results should be considered hypothesis-generating.

## Introduction

Venous thromboembolism (VTE), encompassing deep vein thrombosis (DVT) and pulmonary embolism (PE), is a major global health concern. In Western populations, the annual incidence of VTE is estimated at approximately 1–2 cases per 1,000 person-years (1). The clinical impact of VTE extends beyond the acute event, as it is linked not only to a substantially increased risk of mortality but also to a higher incidence of subsequent arterial cardiovascular events (2). VTE may also result in serious long-term complications, including post-thrombotic syndrome and recurrent events, highlighting the importance of effective preventive strategies (3, 4).

Prior studies have shown that obesity confers more than a twofold higher risk of a first episode of VTE compared with normal weight (5), probably attributable to chronic inflammation and a hypercoagulable state (6). Weight reduction has also been associated with a lower risk of VTE (7, 8). In recent years, glucagon-like peptide-1 receptor agonists (GLP-1 RAs), originally developed for diabetes management, have also demonstrated substantial weight loss benefits (9). Moreover, GLP-1 RAs have been shown to exert anti-inflammatory effects (10), which may contribute to their cardiovascular and renal benefits (11). Observational studies have further suggested that GLP-1 RAs use, compared with dipeptidyl peptidase-4 inhibitors (DPP4is), may be associated with a reduced risk of VTE in individuals with type 2 diabetes (T2D) (12). More recently, tirzepatide, a dual glucose-dependent insulinotropic polypeptide (GIP) and GLP-1 receptor agonist, has been shown in a randomized controlled trial to achieve greater weight reduction than semaglutide (13). In another observational study, tirzepatide demonstrated superior cardiovascular outcomes compared with GLP-1 RAs in patients with T2D (14).

However, evidence remains limited regarding whether tirzepatide use is associated with a reduced incidence of VTE compared with GLP-1 RAs among patients with obesity. To address this knowledge gap, we conducted a real-world cohort study using the TriNetX database to evaluate the comparative effectiveness of tirzepatide versus GLP-1 RAs in reducing the 1-year incidence of VTE in patients with obesity.

## Methods

### Data source

This retrospective cohort study leveraged data from the TriNetX platform, a global federated health research network encompassing electronic health records from roughly 184 million patients across 157 healthcare organizations. The dataset includes information on diagnoses, procedures, medications, laboratory results, and genomic data. Only aggregated counts and de-identified summary statistics are available through TriNetX, with no access to protected health information or study-specific interventions. Accordingly, this study used de-identified data from the TriNetX research network and was exempt from review by the Western Institutional Review Board. The study protocol was reviewed and approved by the Institutional Review Board of Chi Mei Medical Center (IRB No. 11402-E02), with a waiver of informed consent due to the use of anonymized data. The study’s design and reporting adhered to the Strengthening the Reporting of Observational Studies in Epidemiology (STROBE) guidelines (15).

### Study design

We identified adults aged 18 years or older with a documented diagnosis of obesity (ICD-10-CM code E66.0, E66.2, E66.9; or had a document of body mass index ≥30 kg/m^2^) who initiated treatment with either tirzepatide or GLP-1RAs between January 1, 2022, and August 31, 2025 (16, 17). The study population included only new users of the therapies of interest, excluding individuals with any prior or concurrent exposure to these medications before the index date. Patients who had received anticoagulants within the year preceding the index date were also excluded. The index date was defined as the first recorded initiation of the respective therapy. An active-comparator, new-user design was applied, directly comparing tirzepatide initiators with GLP-1RAs initiators to reduce confounding by indication and ensure temporal alignment of baseline characteristics. To capture incident events, individuals with any occurrence of study outcomes prior to the follow-up period were excluded. Both treatment groups were identified within the same study period to minimize temporal differences in patient selection and prescribing patterns. Detailed definitions and coding algorithms for all variables are provided in Supplementary Table 1.

### Covariates and propensity score matching

After defining the study cohorts, index dates, outcomes, and potential confounders, we created a covariate matrix using patient-level information from the 12 months preceding the index date. Propensity scores were calculated using logistic regression to estimate each individual’s probability of initiating tirzepatide based on baseline characteristics. We then performed 1:1 greedy nearest-neighbor matching without replacement, applying a caliper width equal to 0.1 of the pooled standard deviation of the logit of the propensity score. Covariate balance was considered satisfactory when the standardized mean difference (SMD) was less than 0.1 (18).

The selection of baseline covariates was informed by prior literature and clinical relevance, with the aim of capturing key demographic characteristics, comorbid conditions, and laboratory parameters that are known or plausibly associated with both treatment allocation and the risk of venous thromboembolism. These variables were included to reduce confounding and improve comparability between treatment groups (19–23). The propensity score matching (PSM) incorporated age (years, mean [SD]), sex (female, *n* [%]), and race (White and Black or African American, *n* [%]). Comorbidities included hypertension, dyslipidemia, neoplasms, ischemic heart diseases, chronic kidney disease, COVID-19, heart failure, chronic obstructive pulmonary disease, peripheral vascular diseases, systemic involvement of connective tissue, cerebral infarction, other and unspecified cirrhosis of liver, and human immunodeficiency virus disease (all *n* [%]). Laboratory and anthropometric measurements comprised body mass index (kg/m^2^, mean [SD]), estimated glomerular filtration rate (mL/min/1.73 m^2^, mean [SD]), and hemoglobin A1c (%, mean [SD]). Complete definitions and operational codes for all covariates are provided in Supplementary Table 2.

### Outcomes and follow-up

The primary outcome was VTE, with secondary outcomes including DVT, PE, and all-cause mortality. To assess the robustness of the findings, a negative control outcome (skin cancer) was also examined. Outcome follow-up began the day after the index date and continued until the earliest occurrence of the outcome event, the last available clinical encounter, death, or 1 year after the index date.

### Subgroup analysis

For the primary outcome, prespecified subgroup analyses were performed according to sex, age group (18–64 or ≥65 years), and the presence of comorbidities, including neoplasms, atrial fibrillation, chronic kidney disease, and heart failure.

### Statistical analysis

Continuous variables were reported as means with standard deviations, and categorical variables as counts with corresponding percentages. To improve comparability between treatment groups, PSM was applied to balance baseline characteristics before conducting the primary, subgroup, and sensitivity analyses. Hazard ratios (HRs) with 95% confidence intervals (CIs) were estimated using Cox proportional hazards regression models. Time-to-event outcomes were assessed with Kaplan–Meier survival curves and compared using log-rank tests. E-values were additionally calculated to evaluate the potential influence of unmeasured confounding (24). Continuous variables were reported as means and standard deviations as provided by the TriNetX platform; assessment of normality and reporting of medians with interquartile ranges were not available within the analytic interface. All statistical analyses were conducted within the TriNetX analytics platform.

### Sensitivity analyses

Several sensitivity analyses were conducted to assess the robustness of the primary findings. First, stratified analyses were performed according to baseline HbA1c levels (≥7% vs. <7%) to evaluate whether residual imbalance in glycemic control influenced the observed associations. Second, an on-treatment analysis was conducted to better approximate sustained exposure. Given that treatment discontinuation and switching cannot be directly ascertained within the TriNetX platform, patients were required to have continued records of the initial treatment between 3 months and 1 year after the index date. Individuals without evidence of continued use during this period were excluded. Third, additional analyses were performed using alternative comparator definitions, including restriction to semaglutide, to assess the impact of treatment heterogeneity.

## Results

### Study cohort

**Figure 1:**
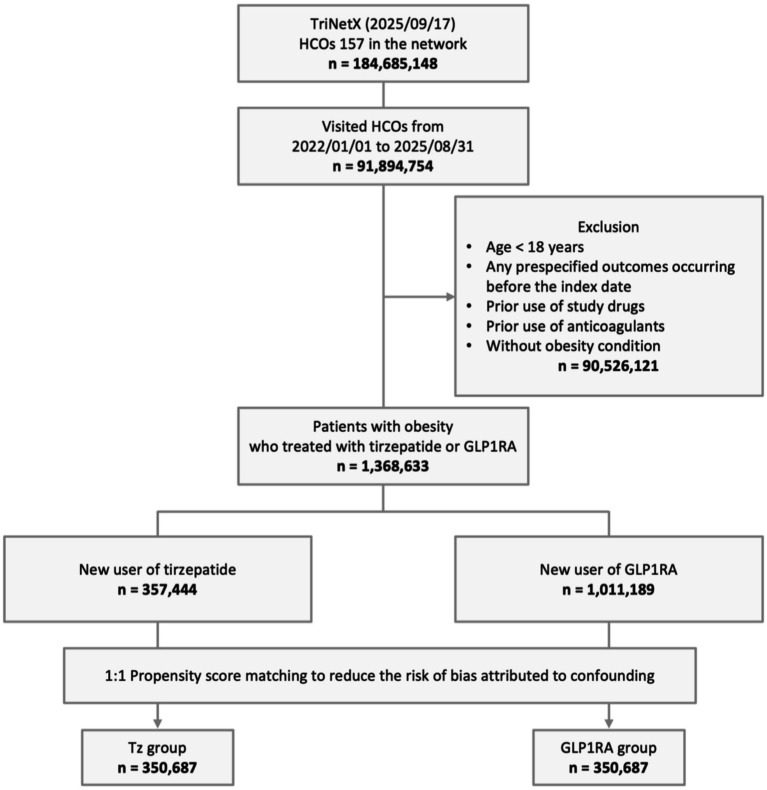
Study cohort process. GLP1RA, glucagon-like peptide-1 receptor agonist; HCOs, healthcare organizations; Tz, tirzepatide.

Among the 184,685,148 patients in the TriNetX network as of September 17, 2025, 91,894,754 had at least one clinical encounter between January 1, 2022, and August 31, 2025. After excluding individuals younger than 18 years, those with any prespecified outcomes before the index date, prior use of study drugs or anticoagulants, and those without obesity, 1,368,633 eligible patients with obesity who were treated with either tirzepatide or GLP-1RAs were identified. Of these, 357,444 were new users of tirzepatide and 1,011,189 were new users of GLP-1RAs. To minimize confounding and improve baseline comparability, 1:1 PSM was performed, resulting in two well-balanced cohorts of 350,687 patients each in the tirzepatide and GLP-1RA groups (Figure 1).

### Characteristics of study subjects

Table 1 presents the baseline characteristics of patients in the tirzepatide and GLP-1RA groups before and after PSM. Prior to matching, patients initiating tirzepatide were slightly younger (mean age 51.3 vs. 52.0 years), more frequently White, and less likely to be Black or African American compared with GLP-1RAs users. Several comorbidities, including neoplasms, chronic obstructive pulmonary disease, and cerebral infarction, were somewhat more prevalent among tirzepatide users, whereas ischemic heart diseases, chronic kidney disease, and heart failure were slightly more common among GLP-1RAs users. The mean HbA1c level was lower in the tirzepatide group (6.6% vs. 7.4%), while body mass index and eGFR were broadly comparable between groups. Following 1:1 PSM, 350,687 well-matched patients remained in each group, achieving excellent covariate balance with standardized mean differences less than 0.1 for nearly all variables. After matching, demographic characteristics, comorbidity profiles, and laboratory measurements were closely aligned, supporting comparability between cohorts for subsequent outcome analyses.

**Table 1:** Baseline characteristics of tirzepatide and GLP1-RAs groups before and after matching.

Variables	Before matching			After matching		
	Tirzepatide group (*n* = 350,689)	GLP-1RAs group (*n* = 1,010,638)	Standardized difference	Tirzepatide group (*n* = 350,687)	GLP1-RAs group (*n* = 350,687)	Standardized difference
Age, years						
Mean (SD)	51.3 (13.6)	52 (14.4)	0.045	51.3 (13.6)	51.1 (13.8)	0.014
Sex, *n* (%)						
Female	232,596 (66.3)	640,036 (63.3)	0.063	232,594 (66.3)	232,659 (66.3)	0
Race, *n* (%)						
White	246,130 (70.2)	642,666 (63.6)	0.14	246,128 (70.2)	245,285 (69.9)	0.005
Black or African American	52,218 (14.9)	194,067 (19.2)	0.115	52,218 (14.9)	52,803 (15.1)	0.005
Comorbidities, *n* (%)						
Hypertension	158,698 (45.3)	466,341 (46.1)	0.018	158,696 (45.3)	156,267 (44.6)	0.014
Dyslipidemia	154,092 (43.9)	426,957 (42.2)	0.034	154,090 (43.9)	150,788 (43)	0.019
Neoplasms	59,516 (17)	143,061 (14.2)	0.078	59,514 (17)	57,042 (16.3)	0.019
Ischemic heart diseases	27,715 (7.9)	90,687 (9)	0.039	27,715 (7.9)	25,103 (7.2)	0.028
Chronic kidney disease	21,629 (6.2)	70,492 (7)	0.033	21,629 (6.2)	19,388 (5.5)	0.027
COVID-19	16,691 (4.8)	44,940 (4.4)	0.015	16,690 (4.8)	15,527 (4.4)	0.016
Heart failure	13,576 (3.9)	45,586 (4.5)	0.032	13,576 (3.9)	11,377 (3.2)	0.034
Chronic obstructive pulmonary disease	10,231 (2.9)	37,146 (3.7)	0.042	10,231 (2.9)	9,285 (2.6)	0.016
Peripheral vascular diseases	5,496 (1.6)	18,577 (1.8)	0.021	5,496 (1.6)	4,677 (1.3)	0.02
Systemic involvement of connective tissue	3,503 (1)	8,068 (0.8)	0.021	3,503 (1)	3,003 (0.9)	0.015
Cerebral infarction	3,494 (1)	13,453 (1.3)	0.031	3,494 (1)	3,044 (0.9)	0.013
Other and unspecified cirrhosis of liver	2,912 (0.8)	9,029 (0.9)	0.007	2,912 (0.8)	2,508 (0.7)	0.013
Human immunodeficiency virus disease	1,190 (0.3)	4,306 (0.4)	0.014	1,190 (0.3)	1,157 (0.3)	0.002
Body mass index, kg/m^2^, Mean (SD)	38.6 (7.3)	38.5 (7.3)	0.013	38.6 (7.3)	38.5 (7.2)	0.015
eGFR, mL/min/1.73 m^2^, Mean (SD)	82.1 (23.4)	81.9 (26.1)	0.008	82.1 (23.4)	82.8 (25.1)	0.03
HbA1c, %, Mean (SD)	6.6 (1.7)	7.4 (2.1)	0.417	6.6 (1.7)	7.3 (2.1)	0.354

eGFR, estimated Glomerular filtration rate; SD, standard deviation.

### Primary and secondary outcomes

**Figure 2:**
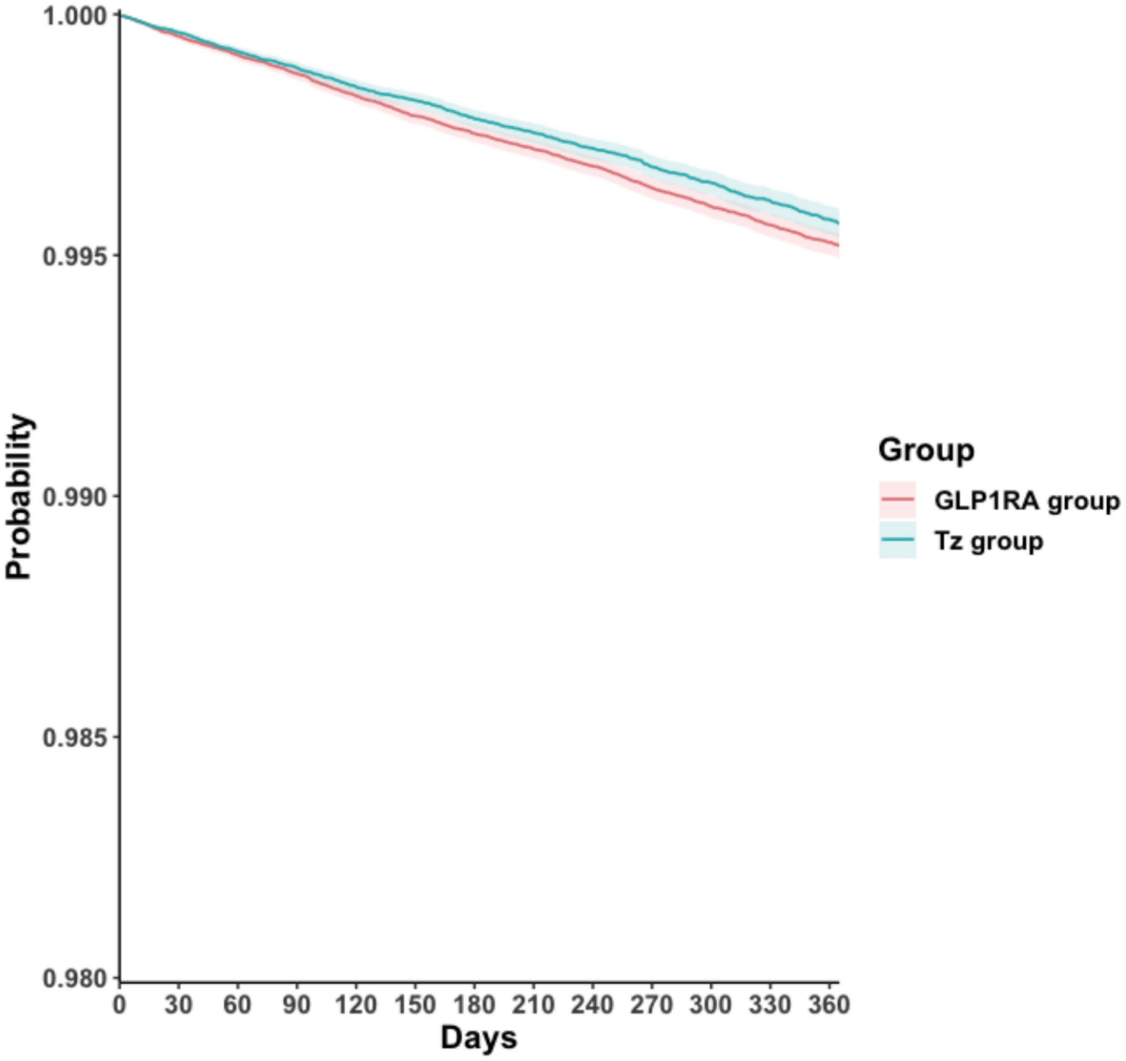
Kaplan–Meier time-to-event free curves of venous thromboembolism comparison to tirzepatide and GLP1-RAs groups. GLP1RA, glucagon-like peptide-1 receptor agonist; Tz, tirzepatide.

Table 2 summarizes the incidence rates, HRs, and E-values for the primary and secondary outcomes after PSM. For the primary outcome, tirzepatide initiation was associated with a significantly lower risk of VTE compared with GLP-1RAs initiation (HR, 0.90; 95% CI, 0.83–0.98; *p* = 0.011). Kaplan–Meier curves demonstrated a consistently higher VTE-free probability in the tirzepatide group than in the GLP-1RAs group (log-rank *p* = 0.011, Figure 2). Regarding secondary outcomes, tirzepatide users exhibited a significantly lower risk of PE (HR, 0.88; 95% CI, 0.79–0.98; *p* = 0.021) and all-cause mortality (HR, 0.90; 95% CI, 0.82–0.99; *p* = 0.024), whereas no significant difference was observed for DVT (HR, 0.96; 95% CI, 0.86–1.07; *p* = 0.410). E-value analyses suggested that the observed associations may be sensitive to unmeasured confounding. To assess the potential impact of residual confounding, we examined a negative control outcome that are not expected to be influenced by the exposure. No significant association was observed between tirzepatide use and the risk of skin cancer (HR, 0.99; 95% CI, 0.93–1.05; *p* = 0.697, Supplementary Table 4).

**Table 2:** Hazard ratio of outcomes between tirzepatide and GLP-1RAs groups.

Outcome	Tirzepatide group (*n* = 350,687)	GLP1-RAs group (*n* = 350,687)	HR (95% CI)	*p* value	E-value (95% LCL)
	Events (%)	Events (%)			
Primary outcome					
VTE	894 (0.3)	1,415 (0.4)	0.90 (0.83,0.98)	0.011	1.46 (1.16)
Secondary outcomes					
DVT	558 (0.2)	823 (0.2)	0.96 (0.86,1.07)	0.410	1.25 (1.0)
PE	516 (0.1)	835 (0.2)	0.88 (0.79,0.98)	0.021	1.53 (1.16)
All-cause mortality	706 (0.2)	1,127 (0.3)	0.90 (0.82,0.99)	0.024	1.46 (1.11)

CI, confidence interval; DVT, deep vein thrombosis; GLP1RA, glucagon-like peptide-1 receptor agonist; HR, hazard ratio; LCL, lower confidence limit; PE, pulmonary embolism; Tz, tirzepatide; VTE, venous thromboembolism.

### Subgroup analysis

**Figure 3:**
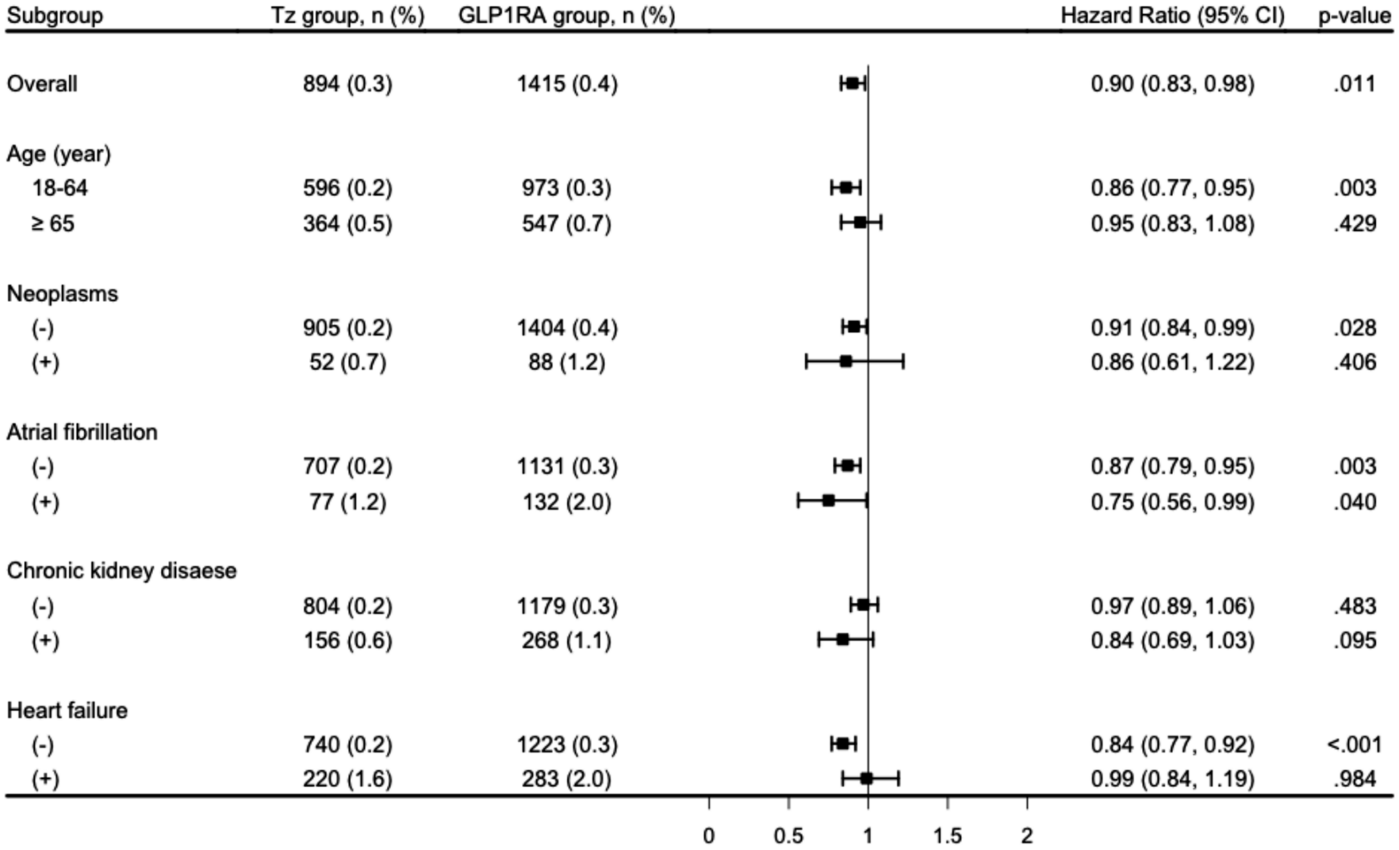
Subgroup analysis for the risk of venous thromboembolism comparison to tirzepatide and GLP1RA groups. CI, confidence interval; GLP1RA, glucagon-like peptide-1 receptor agonist; Tz, tirzepatide.

Figure 3 presents the results of prespecified subgroup analyses for the association between tirzepatide use and the risk of VTE. Across all examined subgroups, the HRs consistently favored tirzepatide, with point estimates below 1.0. The observed associations appeared to be more pronounced among younger patients and those with lower comorbidity burden; however, these findings should be interpreted cautiously, as subgroup analyses were exploratory and not supported by formal interaction testing. Therefore, it remains uncertain whether these differences reflect true effect modification or residual confounding. Similar protective associations were observed in patients with or without atrial fibrillation, indicating that the effect of tirzepatide on VTE risk was broadly consistent across clinically relevant subgroups. Although no statistically significant differences were observed in the subgroups with or without chronic kidney disease or neoplasms, the trends were directionally consistent.

### Sensitivity analysis

Supplementary Table 5 shows the sensitivity analyses for the primary outcome. The primary findings remained generally consistent across multiple approaches. Stratified analyses by baseline HbA1c levels showed similar effect estimates in both subgroups (HbA1c ≥ 7%: HR 0.88 [0.75–1.04]; HbA1c < 7%: HR 0.92 [0.80–1.04]), with no statistically significant differences observed. In addition, an on-treatment analysis yielded a comparable association (HR 0.89 [0.80–0.99]), suggesting that the findings were not substantially influenced by treatment discontinuation or switching. Analyses using alternative comparator definitions, including restriction to semaglutide, produced consistent results (HR 0.99 [0.93–1.10]).

## Discussion

In this large, real-world cohort study of 701,374 patients with obesity, initiation of tirzepatide was associated with a significantly lower 1-year risk of VTE compared with GLP-1 RAs. The benefit was mainly driven by reductions in PE and all-cause mortality, whereas the risk of DVT did not differ significantly between groups. These associations were robust across multiple sensitivity analyses, including negative control outcomes, and were consistent across clinically relevant subgroups, with more pronounced effects in patients aged 18–64 years and those without comorbid conditions such as heart failure. Collectively, these findings suggest a potential association between tirzepatide use and a lower risk of VTE compared with GLP-1 RAs.

In a large real-world cohort study including 540,258 patients with T2D, GLP-1 RAs use was associated with a significantly lower risk of VTE compared with DPP-4 inhibitors (HR 0.78; 95% CI 0.73–0.83). Similarly, a recent meta-analysis of 27 randomized controlled trials involving 84,003 patients reported a trend toward reduced VTE risk with GLP-1 RAs (RR 0.70; 95% CI, 0.46–1.07), with a more pronounced benefit observed for pulmonary embolism (RR, 0.60; 95% CI, 0.39–0.94) in patients with T2D and obesity (25). Our findings align with these observations: tirzepatide was associated with a significant reduction in PE compared with GLP-1 RAs, whereas no significant difference was observed for DVT, although the directional trend was consistent. These findings are broadly consistent with prior literature suggesting a more pronounced association for PE than for DVT. Taken together, these parallels suggest that the protective effect of incretin-based therapies on thromboembolic risk may be driven primarily by reductions in PE rather than DVT. Our findings may be partly explained by the superior cardiometabolic benefits of tirzepatide compared with GLP-1 RAs, including greater weight loss and improved cardiovascular risk profiles (13, 14). However, while prior trials mainly focused on glycemic and cardiovascular outcomes, evidence regarding VTE risk has been limited. Our study therefore provides novel real-world evidence suggesting that tirzepatide may confer additional protection against thromboembolic events beyond that offered by GLP-1 RAs.

The observed association between tirzepatide use and lower VTE risk may be influenced by differences in weight reduction and metabolic improvements between treatment groups (26). However, these factors were not fully captured in the database and may represent sources of residual confounding. Obesity is a well-established risk factor for VTE, conferring more than a twofold higher risk compared with normal weight individuals, largely mediated through chronic inflammation and a hypercoagulable state (27, 28). Weight reduction itself has been associated with a significantly lower risk of VTE (7, 8). Beyond weight loss, tirzepatide has been shown in prior studies to have anti-inflammatory and metabolic effects (29), improve endothelial function, and may attenuate platelet activation (30, 31); however, whether these mechanisms translate into reduced thromboembolic risk remains uncertain.

Prior evidence suggests that the potential association between incretin-based therapies and VTE may be more pronounced for PE than for DVT (12). In a recent meta-analysis of randomized controlled trials, GLP-1 RAs were associated with a greater reduction in PE compared with DVT (25). Several factors may contribute to this difference. PE is often a clinically acute and severe condition, which may be more consistently diagnosed and recorded in electronic health records. In contrast, DVT may be underdiagnosed, particularly in asymptomatic or mildly symptomatic cases, potentially attenuating observed associations in real-world data.

Importantly, this differential ascertainment may introduce detection bias, which could partly explain the observed discrepancy between pulmonary embolism and deep vein thrombosis. As such, this inconsistency may limit the internal validity of the findings and should be interpreted with caution. In addition, differences in underlying pathophysiology between PE and DVT have been proposed, although these mechanisms remain incompletely understood. Therefore, the observed discrepancy should be interpreted with caution and requires further investigation.

This study leveraged a large, multicenter real-world database and employed propensity score matching to strengthen methodological rigor and reduce the influence of measured confounders. In patients with obesity and no prior history of VTE, tirzepatide use was associated with a significantly lower 1-year incidence of VTE, along with reduced risks of PE and all-cause mortality compared with GLP-1 RAs therapy. These findings provide encouraging evidence that tirzepatide may be associated with a lower risk of thromboembolic events. However, given the inherent limitations of observational research, confirmation through randomized controlled trials and long-term outcome studies is essential to validate these associations and to better define their clinical implications.

In the absence of established pharmacologic strategies for the primary prevention of VTE in patients with obesity, our findings suggest that tirzepatide may provide an additional protective benefit beyond its established effects on glycemic control and weight reduction. Current clinical guidelines primarily recommend GLP-1RAs for patients with obesity and T2D based on cardiovascular and metabolic benefits (32, 33); however, their role in VTE prevention has not been defined. By demonstrating a significant reduction in PE and overall VTE risk, our study highlights the potential of tirzepatide to address an important unmet clinical need in this high-risk population. Overall, the findings of this study should be interpreted in the context of several limitations, including residual confounding, potential exposure misclassification, and modest effect sizes. Therefore, the results should be considered hypothesis-generating rather than definitive evidence of a causal relationship. These findings may help generate hypotheses for future studies and may inform the design of prospective trials. Notably, the absolute risk reduction observed in this study was small, which further limits the immediate clinical applicability of these findings.

## Limitations

This study has several limitations. First, as TriNetX is a registry-based database, the risk of misidentification and underrepresentation exists, particularly among individuals with milder disease or limited healthcare utilization, which may affect the generalizability of our findings. In addition, the use of diagnostic codes to define exposures, covariates, and outcomes raises the possibility of misclassification bias. In particular, differences in treatment-related weight loss and metabolic improvements between tirzepatide and GLP-1 RA could not be fully accounted for, which may have influenced the observed associations. To mitigate this concern, we performed negative control outcome analyses with unrelated clinical conditions, which revealed no significant differences between treatment groups, suggesting that bias from diagnostic coding was likely minimal. Additionally, subgroup analyses were exploratory, and formal interaction testing was not performed. Therefore, potential effect modification across patient subgroups cannot be definitively established.

Second, the duration of tirzepatide and GLP-1 RA therapy could not be reliably ascertained from the database, limiting our ability to evaluate the effects of treatment duration or long-term exposure. In addition, calendar time was not explicitly adjusted for in the propensity score model. Given that tirzepatide is a relatively new therapy, prescribing patterns and patient characteristics may have evolved over the study period. Although an active-comparator, new-user design was applied within a shared time frame, residual temporal confounding cannot be excluded and may have influenced the observed associations. Third, despite the inclusion of multiple clinically relevant covariates, some potentially important variables may not have been available or fully captured in the database, which may result in residual confounding. To further explore this possibility, we calculated E-values. The E-values observed in this study were modest in magnitude, suggesting that the observed associations could potentially be explained by relatively weak unmeasured confounding. Therefore, these findings should be interpreted with caution. Fourth, the distribution of continuous variables could not be formally assessed, as the TriNetX platform provides summary statistics primarily as means and standard deviations. Therefore, non-normal distributions may not have been fully characterized. Fifth, death was not treated as a competing risk. Given that all-cause mortality differed slightly between groups, this may have influenced the estimated risk of VTE.

Sixth, residual imbalance in HbA1c persisted after propensity score matching, indicating potential differences in baseline metabolic status between groups. Although sensitivity analyses stratified by HbA1c levels yielded consistent results, residual confounding related to glycemic control and overall metabolic health cannot be excluded. Seventh, GLP-1RAs were analyzed as a class, which may introduce heterogeneity due to differences in pharmacologic profiles and clinical indications across agents. Although sensitivity analyses restricted to semaglutide yielded consistent results, residual heterogeneity and confounding by indication cannot be excluded. Eighth, exposure misclassification is also possible, as treatment adherence, discontinuation, and switching could not be directly assessed within the database. Although an on-treatment analysis was performed, this approach relied on indirect definitions and may not fully reflect actual treatment patterns. Ninth, the PSM approach was subject to limitations related to data availability within the TriNetX platform. Missing data were not explicitly imputed, and analyses were conducted based on available information, which may have influenced cohort selection and matching performance. In particular, laboratory variables such as HbA1c were not available for all patients, and incomplete data may have introduced selection bias or residual confounding. Furthermore, although covariates included in the propensity score model were selected based on clinical relevance and prior literature, the availability of certain variables may have been inconsistent across patients. As a result, the matching process may not have fully accounted for all clinically important differences between groups.

Finally, owing to inherent limitations of the database, specific causes of mortality were unavailable, which may have led to misclassification of clinical endpoints.

## Conclusion

Tirzepatide use was associated with a modestly lower 1-year incidence of VTE compared with GLP-1 receptor agonists in individuals with obesity. However, the absolute risk difference was small, and the clinical significance of this finding remains uncertain.

These results should be interpreted as associative and hypothesis-generating rather than indicative of a causal or clinically actionable effect. Further prospective studies are required to confirm these findings and to determine their potential clinical relevance.

## Data Availability

The data analyzed in this study is subject to the following licenses/restrictions: the data are subject to TriNetX data use agreements and privacy regulations. Individual-level patient data are de-identified and cannot be downloaded or shared. Only aggregated results generated within the TriNetX platform are accessible to investigators. Requests to access these datasets should be directed to https://trinetx.com/.
