# Supplementary material for: Comparative effectiveness of tirzepatide versus GLP-1 receptor agonists on the risk of venous thromboembolism in patients with obesity: a real-world cohort study
Source: Front Med (Lausanne). 2026 May 19;13:1820366. doi: 10.3389/fmed.2026.1820366 (PMC13226182; doi:10.3389/fmed.2026.1820366)
Supplement: Supplementary file 1 [file Data_Sheet_1.DOCX]

**eTable 1.** Demographic, diagnostic, procedural, medication, visit, and laboratory codes used in the definition of the cohorts

| **Category** | **Code** | **Description** |
| --- | --- | --- |
| **Tz group** | | |
| **#1**: At least 18 years old | | |
| Demographics | Age | Age (at least 18 years) |
| **#2**: Patients with obesity who treated with Tz  (#2.1 must be fulfilled after #2.2) | | |
| #2.1: Patients with Tz | | |
| medication | NLM:RXNORM:2601723 | tirzepatide |
| #2.2: Patients with obesity | | |
| diagnosis | UMLS:ICD10CM:E66.0 | Obesity due to excess calories |
| diagnosis | UMLS:ICD10CM:E66.2 | Morbid (severe) obesity with alveolar hypoventilation |
| diagnosis | UMLS:ICD10CM:E66.9 | Obesity, unspecified |
| laboratory | TNX:9083 | BMI (at least 30.00 kg/m2) |
| #3: Visit HCOs since 2022 | | |
| Visit | Visit | Visit more than twice since 2022 |
| #4: Cannot have primary outcome before the index date | | |
| Diagnosis | UMLS:ICD10CM:I26.02 | Saddle embolus of pulmonary artery with acute cor pulmonale |
| Diagnosis | UMLS:ICD10CM:I26.09 | Other pulmonary embolism with acute cor pulmonale |
| Diagnosis | UMLS:ICD10CM:I26.92 | Saddle embolus of pulmonary artery without acute cor pulmonale |
| Diagnosis | UMLS:ICD10CM:I26.93 | Single subsegmental thrombotic pulmonary embolism without acute cor pulmonale |
| Diagnosis | UMLS:ICD10CM:I26.99 | Other pulmonary embolism without acute cor pulmonale |
| Diagnosis | UMLS:ICD10CM:I82.401 | Acute embolism and thrombosis of unspecified deep veins of right lower extremity |
| Diagnosis | UMLS:ICD10CM:I82.402 | Acute embolism and thrombosis of unspecified deep veins of left lower extremity |
| Diagnosis | UMLS:ICD10CM:I82.403 | Acute embolism and thrombosis of unspecified deep veins of lower extremity, bilateral |
| Diagnosis | UMLS:ICD10CM:I82.409 | Acute embolism and thrombosis of unspecified deep veins of unspecified lower extremity |
| Diagnosis | UMLS:ICD10CM:I82.411 | Acute embolism and thrombosis of right femoral vein |
| Diagnosis | UMLS:ICD10CM:I82.412 | Acute embolism and thrombosis of left femoral vein |
| Diagnosis | UMLS:ICD10CM:I82.413 | Acute embolism and thrombosis of femoral vein, bilateral |
| Diagnosis | UMLS:ICD10CM:I82.419 | Acute embolism and thrombosis of unspecified femoral vein |
| Diagnosis | UMLS:ICD10CM:I82.421 | Acute embolism and thrombosis of right iliac vein |
| Diagnosis | UMLS:ICD10CM:I82.422 | Acute embolism and thrombosis of left iliac vein |
| Diagnosis | UMLS:ICD10CM:I82.423 | Acute embolism and thrombosis of iliac vein, bilateral |
| Diagnosis | UMLS:ICD10CM:I82.429 | Acute embolism and thrombosis of unspecified iliac vein |
| #5: Cannot have GLP1RA used | | |
| medication | NLM:ATC:A10BJ | Glucagon-like peptide-1 (GLP-1) analogues |
| #6: Patients without anticoagulants within 1 year before the index date  (#6.1 cannot be fulfilled within 1 year before #2.2) | | |
| #6.1: Patients without anticoagulants | | |
| medication | NLM:RXNORM:1037042 | dabigatran etexilate |
| medication | NLM:RXNORM:11289 | warfarin |
| medication | NLM:RXNORM:1364430 | apixaban |
| medication | NLM:RXNORM:1114195 | rivaroxaban |
| medication | NLM:RXNORM:1546356 | dabigatran |
| medication | NLM:RXNORM:1599538 | edoxaban |
| **GLP1RA group** | | |
| **#1**: At least 18 years old | | |
| Demographics | Age | Age (at least 18 years) |
| **#2**: Patients with obesity who treated with GLP1RA  (#2.1 must be fulfilled after #2.2) | | |
| #2.1: Patients with GLP1RA | | |
| medication | NLM:ATC:A10BJ | Glucagon-like peptide-1 (GLP-1) analogues |
| #2.2: Patients with obesity | | |
| diagnosis | UMLS:ICD10CM:E66.0 | Obesity due to excess calories |
| diagnosis | UMLS:ICD10CM:E66.2 | Morbid (severe) obesity with alveolar hypoventilation |
| diagnosis | UMLS:ICD10CM:E66.9 | Obesity, unspecified |
| laboratory | TNX:9083 | BMI (at least 30.00 kg/m2) |
| **#3**: Visit HCOs since 2022 | | |
| Visit | Visit | Visit more than twice since 2022 |
| #4: Cannot have primary outcome before the index date | | |
| Diagnosis | UMLS:ICD10CM:I26.02 | Saddle embolus of pulmonary artery with acute cor pulmonale |
| Diagnosis | UMLS:ICD10CM:I26.09 | Other pulmonary embolism with acute cor pulmonale |
| Diagnosis | UMLS:ICD10CM:I26.92 | Saddle embolus of pulmonary artery without acute cor pulmonale |
| Diagnosis | UMLS:ICD10CM:I26.93 | Single subsegmental thrombotic pulmonary embolism without acute cor pulmonale |
| Diagnosis | UMLS:ICD10CM:I26.99 | Other pulmonary embolism without acute cor pulmonale |
| Diagnosis | UMLS:ICD10CM:I82.401 | Acute embolism and thrombosis of unspecified deep veins of right lower extremity |
| Diagnosis | UMLS:ICD10CM:I82.402 | Acute embolism and thrombosis of unspecified deep veins of left lower extremity |
| Diagnosis | UMLS:ICD10CM:I82.403 | Acute embolism and thrombosis of unspecified deep veins of lower extremity, bilateral |
| Diagnosis | UMLS:ICD10CM:I82.409 | Acute embolism and thrombosis of unspecified deep veins of unspecified lower extremity |
| Diagnosis | UMLS:ICD10CM:I82.411 | Acute embolism and thrombosis of right femoral vein |
| Diagnosis | UMLS:ICD10CM:I82.412 | Acute embolism and thrombosis of left femoral vein |
| Diagnosis | UMLS:ICD10CM:I82.413 | Acute embolism and thrombosis of femoral vein, bilateral |
| Diagnosis | UMLS:ICD10CM:I82.419 | Acute embolism and thrombosis of unspecified femoral vein |
| Diagnosis | UMLS:ICD10CM:I82.421 | Acute embolism and thrombosis of right iliac vein |
| Diagnosis | UMLS:ICD10CM:I82.422 | Acute embolism and thrombosis of left iliac vein |
| Diagnosis | UMLS:ICD10CM:I82.423 | Acute embolism and thrombosis of iliac vein, bilateral |
| Diagnosis | UMLS:ICD10CM:I82.429 | Acute embolism and thrombosis of unspecified iliac vein |
| #5: Cannot have Tz used | | |
| medication | NLM:RXNORM:2601723 | tirzepatide |
| #6: Patients without anticoagulants within 1 year before the index date  (#6.1 cannot be fulfilled within 1 year before #2.2) | | |
| medication | NLM:RXNORM:1037042 | dabigatran etexilate |
| medication | NLM:RXNORM:11289 | warfarin |
| medication | NLM:RXNORM:1364430 | apixaban |
| medication | NLM:RXNORM:1114195 | rivaroxaban |
| medication | NLM:RXNORM:1546356 | dabigatran |
| medication | NLM:RXNORM:1599538 | edoxaban |

**eTable 2.** Demographic, diagnostic, and laboratory codes used in the definition of covariates

| **Code** | **Description** |
| --- | --- |
| AI | Age at Index |
| 2106-3 | White |
| UNK | Unknown Race |
| F | Female |
| 2054-5 | Black or African American |
| I10 | Essential (primary) hypertension |
| E78 | Disorders of lipoprotein metabolism and other lipidemias |
| I20-I25 | Ischemic heart diseases |
| I50 | Heart failure |
| I73 | Other peripheral vascular diseases |
| N18 | Chronic kidney disease (CKD) |
| J44 | Other chronic obstructive pulmonary disease |
| I63 | Cerebral infarction |
| K74.6 | Other and unspecified cirrhosis of liver |
| C00-D49 | Neoplasms |
| B20 | Human immunodeficiency virus [HIV] disease |
| U07.1 | COVID-19 |
| M35 | Other systemic involvement of connective tissue |
| 8001 | Glomerular filtration rate/1.73 sq M.predicted [Volume Rate/Area] in Serum, Plasma or Blood by Creatinine-based formula (MDRD) |
| 9037 | Hemoglobin A1c/Hemoglobin.total in Blood |
| 9083 | BMI |

**eTable 3.** Diagnostic, visit, and procedural codes used in the definition of outcomes

| **Category** | **Code** | **Description** |
| --- | --- | --- |
| #1: VTE (have any of the following) | | |
| Diagnosis | UMLS:ICD10CM:I26.02 | Saddle embolus of pulmonary artery with acute cor pulmonale |
| Diagnosis | UMLS:ICD10CM:I26.09 | Other pulmonary embolism with acute cor pulmonale |
| Diagnosis | UMLS:ICD10CM:I26.92 | Saddle embolus of pulmonary artery without acute cor pulmonale |
| Diagnosis | UMLS:ICD10CM:I26.93 | Single subsegmental thrombotic pulmonary embolism without acute cor pulmonale |
| Diagnosis | UMLS:ICD10CM:I26.99 | Other pulmonary embolism without acute cor pulmonale |
| Diagnosis | UMLS:ICD10CM:I82.401 | Acute embolism and thrombosis of unspecified deep veins of right lower extremity |
| Diagnosis | UMLS:ICD10CM:I82.402 | Acute embolism and thrombosis of unspecified deep veins of left lower extremity |
| Diagnosis | UMLS:ICD10CM:I82.403 | Acute embolism and thrombosis of unspecified deep veins of lower extremity, bilateral |
| Diagnosis | UMLS:ICD10CM:I82.409 | Acute embolism and thrombosis of unspecified deep veins of unspecified lower extremity |
| Diagnosis | UMLS:ICD10CM:I82.411 | Acute embolism and thrombosis of right femoral vein |
| Diagnosis | UMLS:ICD10CM:I82.412 | Acute embolism and thrombosis of left femoral vein |
| Diagnosis | UMLS:ICD10CM:I82.413 | Acute embolism and thrombosis of femoral vein, bilateral |
| Diagnosis | UMLS:ICD10CM:I82.419 | Acute embolism and thrombosis of unspecified femoral vein |
| Diagnosis | UMLS:ICD10CM:I82.421 | Acute embolism and thrombosis of right iliac vein |
| Diagnosis | UMLS:ICD10CM:I82.422 | Acute embolism and thrombosis of left iliac vein |
| Diagnosis | UMLS:ICD10CM:I82.423 | Acute embolism and thrombosis of iliac vein, bilateral |
| Diagnosis | UMLS:ICD10CM:I82.429 | Acute embolism and thrombosis of unspecified iliac vein |
| #2: All-cause mortality (have any of the following) | | |
| Demographics | Deceased | Deceased |
| Diagnosis | UMLS:ICD10CM:R99 | Ill-defined and unknown cause of mortality |
| #3: PE (have any of the following) | | |
| Diagnosis | UMLS:ICD10CM:I26.02 | Saddle embolus of pulmonary artery with acute cor pulmonale |
| Diagnosis | UMLS:ICD10CM:I26.09 | Other pulmonary embolism with acute cor pulmonale |
| Diagnosis | UMLS:ICD10CM:I26.92 | Saddle embolus of pulmonary artery without acute cor pulmonale |
| Diagnosis | UMLS:ICD10CM:I26.93 | Single subsegmental thrombotic pulmonary embolism without acute cor pulmonale |
| Diagnosis | UMLS:ICD10CM:I26.99 | Other pulmonary embolism without acute cor pulmonale |
| #4: DVT (have any of the following) | | |
| Diagnosis | UMLS:ICD10CM:I82.401 | Acute embolism and thrombosis of unspecified deep veins of right lower extremity |
| Diagnosis | UMLS:ICD10CM:I82.402 | Acute embolism and thrombosis of unspecified deep veins of left lower extremity |
| Diagnosis | UMLS:ICD10CM:I82.403 | Acute embolism and thrombosis of unspecified deep veins of lower extremity, bilateral |
| Diagnosis | UMLS:ICD10CM:I82.409 | Acute embolism and thrombosis of unspecified deep veins of unspecified lower extremity |
| Diagnosis | UMLS:ICD10CM:I82.411 | Acute embolism and thrombosis of right femoral vein |
| Diagnosis | UMLS:ICD10CM:I82.412 | Acute embolism and thrombosis of left femoral vein |
| Diagnosis | UMLS:ICD10CM:I82.413 | Acute embolism and thrombosis of femoral vein, bilateral |
| Diagnosis | UMLS:ICD10CM:I82.419 | Acute embolism and thrombosis of unspecified femoral vein |
| Diagnosis | UMLS:ICD10CM:I82.421 | Acute embolism and thrombosis of right iliac vein |
| Diagnosis | UMLS:ICD10CM:I82.422 | Acute embolism and thrombosis of left iliac vein |
| Diagnosis | UMLS:ICD10CM:I82.423 | Acute embolism and thrombosis of iliac vein, bilateral |
| Diagnosis | UMLS:ICD10CM:I82.429 | Acute embolism and thrombosis of unspecified iliac vein |

**eTable 4**. Negative outcome

| Outcome | HR (95% CI) | *P* value |
| --- | --- | --- |
|  |  |  |
| Skin cancer | 0.988 (0.930,1.050) | 0.697 |

**eTable 5**. Sensitivity analysis for primary outcome

| Outcome | HR (95% CI) | *P* value |
| --- | --- | --- |
|  |  |  |
| HbA1c level |  |  |
| ≥ 7 % | 0.88 (0.75,1.04) | 0.123 |
| < 7% | 0.92 (0.80,1.04) | 0.179 |
| Different Comparator |  |  |
| Tirzepatide vs. Semaglutide | 0.99 (0.93,1.10) | 0.754 |
| On-treatment | 0.89 (0.80,0.99) | 0.034 |
